# SENSory re-learning of the UPPer limb (SENSUPP) after stroke: development and description of a novel intervention using the TIDieR checklist

**DOI:** 10.1186/s13063-021-05375-6

**Published:** 2021-07-05

**Authors:** Håkan Carlsson, Birgitta Rosén, Anders Björkman, Hélène Pessah-Rasmussen, Christina Brogårdh

**Affiliations:** 1 Department of Neurology, Rehabilitation Medicine, Memory Disorders and Geriatrics, Skåne University Hospital, Malmö, Sweden; 2 Department of Health Sciences, Lund University, Lund, Sweden; 3 Department of Translational Medicine – Hand Surgery, Skåne University Hospital, Malmö, Sweden; 4 Department of Hand Surgery, Institute of Clinical Sciences, Sahlgrenska Academy, University of Gothenburg, Gothenburg, Sweden; 5 Department of Clinical Sciences, Lund University, Lund, Sweden

**Keywords:** Sensory training, Stroke, Task-specific training, Upper limb

## Abstract

**Background:**

Sensorimotor impairments of upper limb (UL) are common after stroke, leading to difficulty to use the UL in daily life. Even though many have sensory impairments in the UL, specific sensory training is often lacking in stroke rehabilitation. Thus, the aim of this paper is to provide a detailed description of the novel intervention “SENSory re-learning of the UPPer limb after stroke (SENSUPP)” that we have developed to improve functioning in the UL in persons with mild to moderate impairments after stroke.

**Methods:**

The SENSUPP protocol was designed using information from literature reviews, clinical experience and through consultation of experts in the field. The protocol integrates learning principles based on current neurobiological knowledge and includes repetitive intensive practice, difficulty graded exercises, attentive exploration of a stimulus with focus on the sensory component, and task-specific training in meaningful activities that includes feedback. For reporting the SENSUPP protocol, the Template for Intervention Description and Replication (TIDieR) checklist was used.

**Results:**

The essential features of the SENSUPP intervention comprise four components: applying learning principles based on current neurobiological knowledge, sensory re-learning (exercises for touch discrimination, proprioception and tactile object recognition), task-specific training in meaningful activities, and home-training. The training is performed twice a week, in 2.5-h sessions for 5 weeks.

**Conclusion:**

Since there is close interaction between the sensory and motor systems, the SENSUPP intervention may be a promising method to improve UL functioning after stroke. The TIDieR checklist has been very useful for reporting the procedure and development of the training.

**Trial registration:**

ClinicalTrials.govNCT03336749. Registered on 8 November 2017.

**Supplementary Information:**

The online version contains supplementary material available at 10.1186/s13063-021-05375-6.

## Background

Stroke is a leading cause of adult disability worldwide [1]. Among a variety of impairments following stroke, sensorimotor impairments of the upper limb (UL) are common. Traditionally, most focus in stroke rehabilitation is on recovery of motor function. However, it has been shown that as many as 50% of persons with stroke have sensory impairments of the UL both in the subacute phase [2] and in the chronic phase [3]. Sensory impairments are associated with reduced or prolonged recovery of motor function [4, 5] and are a contributing factor to the development of a lesser spontaneous use of the UL [6]. This negatively affects the ability to use the UL in everyday activities [7], resulting in decreased participation and quality of life [8]. Despite this, assessments of sensory impairments [9], using standardized and reliable outcome measures, are not implemented in the routine care of stroke patients [6]. Persons with stroke also express that sensory training of the hand is often neglected in their rehabilitation [10, 11].

Previous literature describes two different approaches to sensory training for the UL after stroke: passive sensory training and active sensory training [12, 13]. Passive sensory training includes electrical [14] and thermal stimulation [15], whereas active sensory training includes active manual exploration in order to stimulate different sensory modalities. A recent review reported favorable results of passive sensory training [16]. Due to variations in research design and outcome measures, the evidence for active sensory training is still limited [12, 16]. However, Carey et al [17] have shown significant improvement in the ability to discriminate touch from an active sensory training approach, comprising tasks focusing on texture discrimination, limb position sense, and tactile object recognition. Whether active sensory training can lead to an improved ability to use the UL in daily life has not been evaluated and needs to be further explored.

Furthermore, there is evidence that repetitive, task-specific training including intermittent feedback [18, 19] is beneficial for improving motor function of the UL after stroke. Since there is evidence that the sensory and motor systems closely interact [6], there is reason to assume that a combination of sensory and motor training can lead to an improved UL function in persons with stroke. Yet, few studies have however evaluated the effect of combined sensory and motor training [20, 21] for the UL after stroke. Therefore, we have started a pilot randomized controlled trial (pilot RCT) and designed a novel protocol for sensorimotor training of the UL, i.e., the “SENSory re-learning of the UPPer limb after stroke (SENSUPP).” The goal of the SENSUPP study is to evaluate if sensory re-learning in combination with task-specific training is more beneficial than task-specific training alone to improve functioning of UL in persons with mild to moderate stroke [22].

In many RCTs, the intervention is poorly described regarding the training material, number of sessions, duration, dose, intensity and mode of training delivered, etc. [23]. A detailed description of the key components of an intervention would make it easier for other researchers to replicate the intervention in clinical settings and in research [24–26]. Thus, the aim of this paper is to provide a detailed description of the novel intervention in the SENSUPP study using the 12-item Template for Intervention Description and Replication (TIDieR) checklist [23].

## Methods

The SENSUPP study is an ongoing single-blinded pilot RCT with two treatment arms, in which persons with sensory impairment of the UL after stroke are randomized to either sensory re-learning in combination with task-specific training (experimental group) or to task-specific training only (control group; with no focus on sensory re-learning) [22]. In this paper, the intervention for the experimental group is described in detail.

## Results

Description of the SENSUPP protocol

- ***Item 1.*** *Brief name:* Sensory re-learning in combination with task- specific training after stroke
- ***Item 2.*** *Why: Rationale for the intervention*

Stroke often leads to a disorder of the sensory and motor neurons in the central nervous system leading to reduced movement control. The sensory system (including cutaneous mechanoreceptors, joint and muscle proprioceptors as well as vision) is important for the planning of a movement (the feedforward system), for the feedback control of a movement and for motor learning. Hence, an impaired sensory function of the UL after stroke disrupts the normal feedback to the motor network [27], which can affect the movement control of reaching and grasping [28] and force regulation during pinch grip [29] as well as dexterity [30].

In addition, an improved sensory function has proven to be essential for fine motor skills both in healthy subjects [31] and in persons with stroke [32, 33]. Therefore, it may be important to include not only motor training (i.e., task-specific training) in the rehabilitation of the UL after stroke, but also specific sensory training (i.e., sensory re-learning) for persons with sensorimotor impairments. Our hypothesis in the SENSUPP study is that a combination of sensory re-learning and motor training (i.e., task-specific training) is more beneficial than task-specific training alone to improve the sensory function of the hand, dexterity, ability to perform daily hand activities, perceived participation, and life satisfaction.

**Fig. 1:**
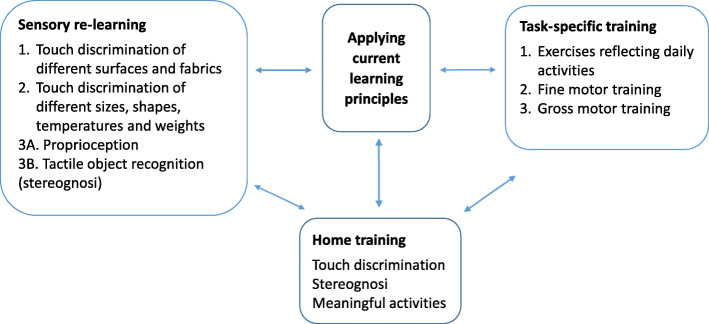
A description of the components in the SENSUPP study

The intervention in the SENSUPP study comprises four components: applying learning principles based on current neurobiological knowledge, sensory re-learning, task-specific training, and home-training (see Fig. 1). The different components of the intervention are described below.

### Applying learning principles

All components in the SENSUPP training are based on current neurobiological learning principles [27]. Important elements to promote learning are, among others, repetitive and intensive practice [34, 35] of various tasks [36], graded exercises of increasing difficulty [18], and attentive exploration of a stimuli with focus on the sensory component [17, 37]. During the training, the participants should concentrate on their sensory impairment, the ability to form an appropriate grasp, the regulation of the grip force, and the ability to perform a daily task. Intrinsic feedback (i.e., the person’s own perceptual feedback while performing a task) is important, which can be obtained by either sensory or visual information or by using the unaffected hand. Extrinsic feedback in terms of verbal and manual guidance from the therapist should also be given [38].

### Sensory re-learning

The sensory re-learning is based on active hand movements that aim to explore properties of various objects. Active hand movements have been found to activate the primary sensory cortex (S1 area) more than passive movements [17, 39], and an integration of sensory and motor stimuli of the hand is essential for exploring and manipulating objects [28, 40]. Thus, the SENSUPP protocol is based on an active sensory training approach including motor training and principles of learning [41]. The sensory re-learning is influenced by post-stroke sensory discrimination training developed by Carey et al. [17] and by the sensory re-learning program developed for persons with peripheral nerve injuries [42]. The main elements for such programs are exercises for touch discrimination, proprioception and tactile object recognition.

### Task-specific training

Task-specific training (i.e., performance of a specific, meaningful and functional task) [43] is recommended to be included in rehabilitation of UL after stroke [44, 45]. The training includes person-centered intensive, repetitive and varied practice [19, 37, 46] with intermittent feedback [47]. The training is performed as “whole reach-to-grasp task” or broken down to “part of the whole task” depending on the participants’ sensorimotor capacity. During the training, the participants are encouraged to concentrate on their sensation and, if possible, to perform the task without vision in order to challenge their sensory function.

### Home training

In order to increase the amount of exercising and to learn to use the affected hand during daily life, the participants are encouraged to train daily at home for 30 min. The training consists of touch discrimination of various textures, fabrics, shapes, and sizes and tactile object recognition [42]. The participants are also encouraged to perform a meaningful task they perceive problematic and wish to improve. In all exercises, participants are encouraged to concentrate on their sensation, the object’s properties, and the performance.

- ***Item 3.*** *What: Materials used in the intervention*

All materials were purchased from commercial companies available through web sites (see Additional file 1 for further details about the addresses).

### Material for the sensory re-learning

**Fig. 2:**
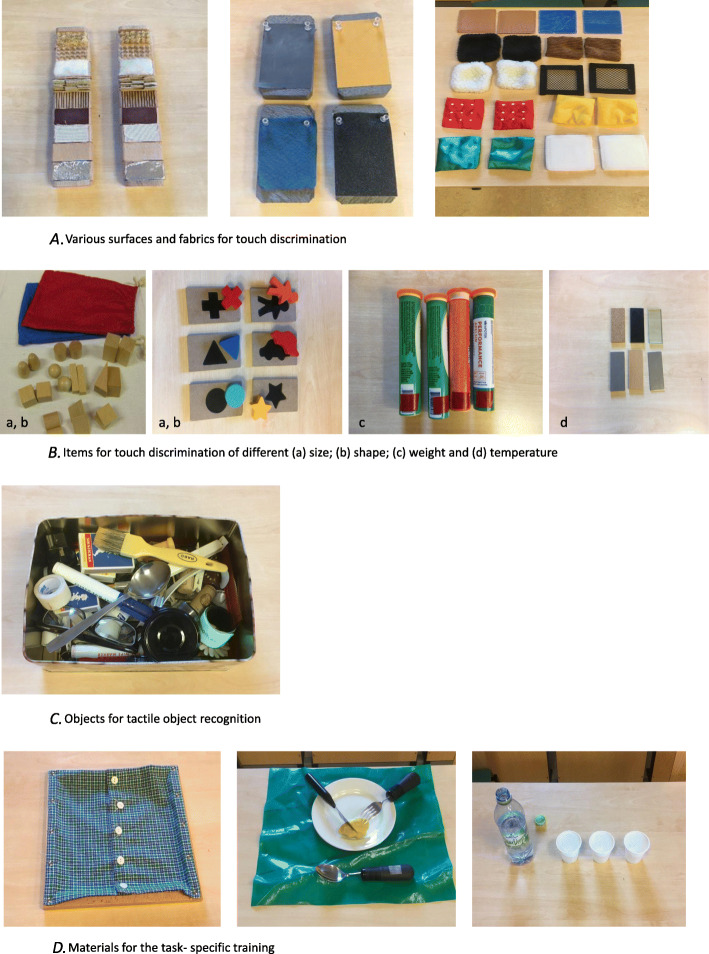
**A** Various surfaces and fabrics for touch discrimination. **B** Items for touch discrimination of different (a) size; (b) shape; (c) weight and (d) temperature. **C** Objects for tactile object recognition. **D** Materials for the task- specific training

The material used for the touch discrimination training, i.e., touching and exploring different surfaces and fabrics with one or several fingers are small circular objects 3 cm in diameter and rectangular 5 × 3 cm to 12 × 8 cm dimensions. Both smooth and rough materials are used, as well as slippery and non-slippery surfaces and fabrics. For examining objects with the whole hand, different 10 × 10 cm fabrics and materials such as leather, plastic, fleece, silk, and net in a size of 8 × 8 cm are used (Fig. 2A).

The material used for discrimination between different sizes and shapes are two and three-dimensional objects, geometric figures in wood of different sizes. In order to practice to discriminate between objects of different weights, participants use weighted tubes of 20 g, 45 g, 70 g, and 95 g. To practice temperature discrimination, 10 × 4 cm plates of stone, cork, glass, metal, wood, and felt are used (Fig. 2B).

The material used for tactile object recognition are ordinary daily objects of varying sizes and materials, such as coins, dices, erasers, safety pins, nuts, screws, thread rolls, pencils, clips, pearls, keys, cutlery, coffee cups, tennis balls, bottle caps, jar caps, rubber bands, toothbrushes, combs, and brushes (Fig. 2C).

### Materials for task-specific training

Materials used for the task-specific training are as follows: shoelaces, a piece of textile with small and large buttons, a textile with a zipper, an assortment of nuts and bolts, a fork and knife to cut off pieces of playdough, a drinking glass, a plastic bottle of 0.5 l to pour water from, and jar lids of various sizes to screw on and unscrew. Other materials are playing cards to sort and deal out, plastic cards in a wallet to remove and replace wooden pegs of different sizes to be inserted into a pegboard placed on a table, or hung on a wall, and objects of varying sizes and weights such as a paper clip, eraser, plastic mug, tape, glass, and weights 0.5–1 kg to move on shelves of different heights (Fig. 2D).

### Materials for home training

The materials used for home training, i.e., for touch discrimination and tactile object recognition are described in the “Material for the sensory re-learning” section.

- ***Item 4.*** *What: Activities and procedures used in the intervention*

### Sensory re-learning

**Fig. 3:**
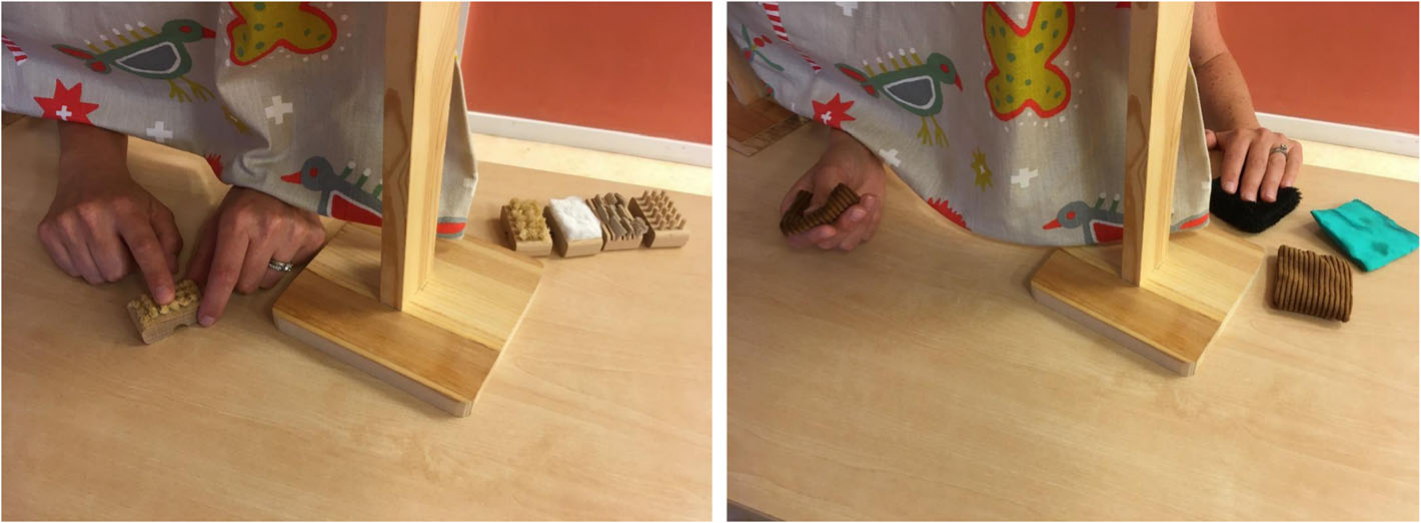
Illustration of sensory re-learning, i.e., examination with the affected hand without the aid of vision

The sensory re-learning starts with the participants sitting at a table in an ergonomic position with the feet on the floor and the arms supported on the table. First, the participants examine the objects with the affected hand behind a curtain without the aid of vision (Fig. 3). Thereafter, they examine the object with the unaffected hand, and finally they examine it with the affected hand while looking at it. To increase the training difficulty, the number of objects is gradually increased and various roughness of the objects’ surfaces and materials are used. The participants are encouraged to concentrate on their sensation and on the characteristics of the objects.

The sensory re-learning comprises:

- 1. *Touch discrimination for exploring different surfaces and fabrics*. The participants are blindfolded and the training starts by examining rougher surfaces and fabrics and is individually progressed to smoother surfaces and fabrics. If necessary, the therapist initially guides the movement passively. Calibration of the surfaces and fabrics is performed through vision or by the non-affected hand.
- 2. *Touch discrimination for identifying objects* with different characteristics such as size, shape, weight, and temperature. The participants are blindfolded and the training starts with a manual exploration of the objects. The difficulty is increased by going from larger to smaller objects. Calibration of the different characteristics is performed through vision or by the non-affected hand.
- 3A. *Proprioception for recognizing the position* of the UL. The training is performed in two different exercises. First, the therapist places the participant’s affected thumb in different positions and asks him/her to locate the thumb with the non-affected hand. Thereafter, the therapist places the participant’s affected UL in different positions and asks the person to place the non-affected UL in the same position.
- 3B. *Stereognosis (tactile gnosis) for identifying various everyday objects*. The participants are blindfolded and requested to recognize and describe what object they are given in their hand. They should manipulate the object in their hand, and if they have difficulties to recognize it, they are encouraged to describe the different properties of the object regarding size, shape, material, and temperature. The participants start with a larger object and gradually progress to smaller objects.

### Task-specific training

In the task-specific training, the selection of reaching- and grasping exercises are based on the participants’ goals and their sensory and motor capacity. The exercises include both fine and gross motor training in various activities such as:

1. *Exercises that reflect daily activities* for example tying shoelaces, doing buttons, pulling up a zipper and using cutlery, assembling and disassembling various nuts and bolts, and putting on and removing a bottle cap and jar lid, as well as pouring water into and out of a cup or bottle.
2. *Fine motor training* for example picking up coins, buttons, clips, and nuts from cans or a flat surface, stacking wooden rods, picking up pegs one at a time and placing them in a pegboard, shuffling, dealing and turning cards, moving coins and marbles from the palm to the fingertip, and manipulating two spheres in the hand.
3. *Gross motor trainin*g for example reaching and moving objects up and down shelves at different heights using various grasps depending on the object's weight, size, and shape and throwing a tennis ball to the floor or against a wall and catching it again with the affected hand.

In all tasks, the participants are encouraged to concentrate on their sensation and on the characteristics of the objects and, if possible, to perform the exercises without vision to challenge the sensory system.

### Home training

The home exercises either consist of tasks focusing on touch discrimination or object recognition depending on the participants’ sensory impairments and goals. Participants are also encouraged to think of the object’s properties carefully when they are using the affected hand in daily activities.

- ***Item 5.*** *Who: Description of the expertise, background, and training of the therapists*

Two skilled physiotherapists with long experience of UL rehabilitation after stroke are involved in the training. Before the start of the intervention, Professor Leeanne Carey (occupational therapist at La Trobe University Melbourne, Australia) with specific expertise of sensory re-learning for persons with stroke was consulted. Thereafter, a training protocol was developed and discussed in the research group. One of the co-workers (BR) demonstrated the training techniques and the two therapists involved in the training discussed the sensory re-learning principles before the trial started. Both physiotherapists participated in the first sessions to become familiar with the training protocol and to discuss how the training could be adapted to the participants’ sensorimotor capacity and goals. Thereafter, only one physiotherapist at a time supervises the training.

- ***Item 6.*** *How: Mode of intervention*

The training is individually adapted and provided in groups of two participants supervised by a physiotherapist.

- ***Item 7.*** *Where: Location where the intervention was given*

The training is conducted in an outpatient clinic at Skånes University Hospital in Sweden. Most of the training is performed in a quiet room without any disturbing activities around.

- ***Item 8.*** *When and how much of the intervention*

The training is performed twice a week for 5 weeks, in 2.5 h sessions, in total 25 h of training [17]. Each training session is comprised of 60 min sensory re-learning, a 15-min break, and 60 min of task-specific training. Each session is in turn, divided into three 20-min blocks during which 2–4 tasks are performed in each block. The physiotherapist records the participant’s training in a protocol. Repeated exercise of each task is important, but the actual number of repetitions for each participant varies depending on his/her capacity and ability to apply the training and learning principles. Every week the participants receive new home-training tasks.

- ***Item 9.*** *Tailoring: Individualizing the intervention*

The training is individually tailored with the ambition to find the optimal training level for each participant. Progression of the difficulty level of the exercises is based on the participants’ sensorimotor capacity, goals, and degree of improvement. The training should be meaningful for the participant and challenging enough to optimize learning.

- ***Item 10.*** *Modifications during the course of study*

Only minor (negligible) modifications of the training protocol have been made so far. Evaluation of the SENSUPP study will add knowledge and offer guidance if the protocol requires modification.

- ***Item 11.*** *Planned procedures for how adherence or fidelity is assessed*

If participants miss one or several training sessions, additional training opportunities are arranged in connection with their 5-week training period to ensure 25 h of training in total. Adherence to the home training is followed-up verbally without any logbook. In order to capture the participants' experiences of the training protocol and possible effects, they are interviewed at 3-month follow-up by an independent therapist not involved in the training.

- ***Item 12.*** *How well the intervention is actually delivered*

So far, the training including number of sessions has successfully been delivered to all participants.

## Discussion

Performance of everyday tasks with one´s hands requires an efficient sensorimotor integration. Since sensory impairments of the UL are common after stroke and specific sensory training is limited in rehabilitation, we developed a novel protocol where we combined sensory re-learning and task-specific training for persons with mild to moderate stroke. Overall, the training is based on current neurobiological learning principles including repetitive, intensive, and varied practice with increasing difficulty and intermittent feedback.

The main elements in the SENSUPP protocol are applying current learning principles, sensory re-learning (exercises for touch discrimination, proprioception and tactile object recognition), task-specific training in meaningful activities, and home-training. A skilled physiotherapist supervises the outpatient training, and to increase the amount of training and to transfer skills into daily practice, home training is also performed.

The training is individually adapted based on the participants’ sensorimotor capacity and goals, and conducted in small groups. One advantage with group training is that the participants can support each other while training and that the training is less time-consuming for the therapist. However, to perform the training in groups could be challenging for the therapist when it comes to individualizing the training to each participant’s capacity and goal and to give adequate feedback on the performance.

Only minor changes of the SENSUPP protocol have been done so far. However, since this is an ongoing pilot RCT, there may be some further additional changes in the training protocol after completion and evaluation of the trial. The results from the SENSUPP study will add new knowledge about the feasibility and effectiveness of sensory re-learning in combination with task-specific training on UL functioning after stroke. It may also contribute to an increased understanding of how the participants perceive the sensory training of the affected hand and the effects of training. In an upcoming study, we will investigate how the participants perceive the training and possible effect. If the new training approach seems to be effective, results from the SENSUPP study can provide knowledge on how to design a larger RCT in persons with sensory impairments of the UL after stroke. A larger RCT is important to be able to fully evaluate and validate the clinical effects of the SENSUPP protocol. For providing a detailed reporting of the development and description of the training in the SENSUPP study, the TIDieR checklist [23] has been very helpful.

## Conclusion

The SENSUPP protocol includes both sensory re-learning and task-specific exercises based on current learning principles. The TIDieR checklist has been very useful to thoroughly describe the training protocol in order to improve the reporting of the development and the procedure of the training. By reporting a detailed description of an intervention, the protocol can be easier replicated by others in clinical practice and in research.

## Supplementary Information

**Additional file 1.**

## Data Availability

Data sharing is not applicable to this article as no datasets were generated or analyzed during the current study.

## Abbreviations

UL: Upper limb
RCT: Randomized controlled trial
SENSUPP: SENSory re-learning of the UPPer limb
TIDieR: Template for Intervention Description and Replication checklist
