# Supplementary material for: SENSory re-learning of the UPPer limb (SENSUPP) after stroke: development and description of a novel intervention using the TIDieR checklist
Source: Trials. 2021 Jul 5;22:430. doi: 10.1186/s13063-021-05375-6 (PMC8259306; doi:10.1186/s13063-021-05375-6)
Supplement: Supplementary file 1 — Additional file 1. [file 13063_2021_5375_MOESM1_ESM.docx]

**Additional file 1**

Web addresses to companies where materials were purchased.

[www.procare.dk](http://www.procare.dk)

[www.lekma.se](file:///C:\Users\ha0783ca\AppData\Roaming\Microsoft\Word\www.lekma.se)

[www.montessorimaterial.nu](file:///C:\Users\ha0783ca\AppData\Roaming\Microsoft\Word\www.montessorimaterial.nu)

[www.komikapp.se](file:///C:\Users\ha0783ca\AppData\Roaming\Microsoft\Word\www.komikapp.se)

[www.invictaeducationshop.com](file:///C:\Users\ha0783ca\AppData\Roaming\Microsoft\Word\www.invictaeducationshop.com)
